# Case report: A new treatment for restless leg syndrome: three cases

**DOI:** 10.3389/fnins.2024.1333188

**Published:** 2024-01-19

**Authors:** Ying Li, Wenjing Zhang, Hui Wang, Weiwei Zhang

**Affiliations:** Third Hospital of Shanxi Medical University, Shanxi Bethune Hospital, Shanxi Academy of Medical Sciences, Tongji Shanxi Hospital, Taiyuan, China

**Keywords:** stellate ganglion block, restless leg syndrome, kidney failure, sympathetic nerve, sleep monitoring report

## Abstract

Restless legs syndrome is a movement disorder that seriously affects the quality of life of patients. It is characterized by marked discomfort mainly occurring in the deep tissues of the lower extremities, including deep muscle or bone chafing, as well as crawling sensations or pulling sensations. These sensations often cause patients to awaken after falling asleep and to feel the urge to walk around, which seriously affects their sleep quality. Patients with restless leg syndrome exhibit significantly enhanced sympathetic nerve activity and immune disorders, while stellate ganglion blockage can block sympathetic nerves and regulate immune cells and cytokines to maintain immune system homeostasis. We report three patients with restless legs syndrome complicated with severe nephrotic syndrome. After treatment with stellate ganglion block, the symptoms in the restless legs were relieved within 1 month, and the quality of sleep was significantly improved. Our findings suggest that stellate ganglion block has broad promise in the management of restless legs syndrome patients with severe comorbidities.

## 1 Introduction

Restless legs syndrome (RLS) is a sensorimotor disease of the nervous system, with an overall prevalence of up to 10% (Ferini-Strambi and Manconi, 2009), and it especially has a high incidence in end-stage renal disease populations. It is characterized by a spontaneous, unbearable painful sensation or abnormal sensations such as ant-crawling and burning sensations, which are more common in the lower limbs and can appear or become aggravated at rest and at night (Trenkwalder et al., 2005). The main goals of treatment for RLS are to relieve symptoms and improve sleep quality. Clinically, dopamine receptor agonists are often used as representative drug treatments; however, when they are used at low doses in the short term, they can quickly relieve symptoms but usually lead to worsening of disease severity (Anguelova et al., 2018). Alternative treatments, such as exercise training, light therapy, and acupuncture, have been shown to be effective at relieving RLS symptoms, but these treatments are long, causing the symptoms to respond slowly (Xu et al., 2018). Therefore, it is necessary to find suitable alternative treatments.

The pathogenesis of RLS is complex and involves various factors, such as brain iron deficiency, altered dopaminergic neuron transmission, peripheral neuropathy, chronic inflammation, and immune deficiency (Weinstock et al., 2012; Allen, 2015; Didato et al., 2020). In recent years, studies have shown that the development of RLS is associated with excessive activation of sympathetic nerves (Stevens, 2015; Bergmann et al., 2021), and inflammation and immune changes are common in RLS patients (Weinstock et al., 2012). Stellate ganglion block (SGB) is a safe and effective minimally invasive treatment. With the innovation of ultrasound monitoring and guidance technology, the safety of SGB can be guaranteed (Narouze, 2014). SGB is mainly used to treat pain, stress, and sleep disorders by blocking sympathetic nerves (Liao et al., 2016; Gu et al., 2022). Moreover, SGB can play a role in regulating immune responses by blocking the sympathetic innervation of immune organs (Lipov et al., 2020). Therefore, we hypothesized that SGB might be effective at relieving RLS symptoms. Relevant research evidence from domestic and foreign studies is insufficient, and there is no experimental evidence supporting the possible therapeutic effect of SGB on RLS.

We report three cases of RLS with severe nephrotic syndrome diagnosed by the updated International Restless Legs Syndrome Study Group (IRLSSG) consensus criterion (Allen et al., 2014). The patients experienced remission after treatment via SGB and experienced few recurrences during follow-up. These observations suggest that the use of stellate ganglion blockade in RLS may be of great clinical value.

## 2 Case report

### 2.1 Case 1

A 47-year-old male, was admitted to the Nephrology Department of our hospital in August 2021 due to dysfunction of the left forearm due to an arteriovenous fistula. He had a history of chronic kidney disease for more than 20 years and had been diagnosed with focal segmental glomerulosclerosis in 2004. He was treated with hormones and immunosuppressants for more than 10 years, during which his creatinine increased progressively. In 2019, he progressed to the uremia stage of chronic renal failure, and his condition was stable after regular hemodialysis. After being admitted to the hospital this time, the doctor observed that his mental state was not good. By careful questioning, during the past 2 years, he had frequently experienced spontaneous shaking of his legs after falling asleep, causing him to awaken. The symptoms were slightly relieved by his arising and engaging in physical activity. The average frequency of the attacks was approximately 5–7 times per hour. The attacks also occurred during the day, but the symptoms were milder than those at night. These symptoms seriously affected the patient’s work efficiency during the day and quality of sleep at night. The effect of dopamine drugs was not good, so the drug was stopped by the patient on his own volition. After a comprehensive evaluation by nephrologists and anesthesiologists, the patient was diagnosed with RLS with an International Registry Sleep Scale (IRLS) score of 34, and he was given SGB therapy once every other day for a total of 6 treatments. The above symptoms basically disappeared 1 month after the treatment, and the IRLS score decreased to 8. To date, only occasional spontaneous shaking of the legs has been observed during exertion, with an IRLS score of 10. In addition, a polysomnography evaluation revealed that the patient’s sleep quality improved significantly after treatment; his episodes of snoring, airflow limitation, and paradoxical breathing were reduced; and the longest sleep pause time was also significantly reduced.

### 2.2 Case 2

A 57-year-old male, was admitted to our hospital in July 2021 due to the need for hemodialysis. The patient was diagnosed with nephrotic syndrome in 2020. After onset, he was treated with hormones and cyclosporine combined with symptomatic treatments such as acid suppression, calcium supplementation, and immune regulation. However, the disease still progressed, but the symptoms were relieved after regular hemodialysis. The patient had been experiencing frequent twitching symptoms in both calves since January 2020, mostly before he fell asleep, which could be immediately relieved by standing. The frequency of falls was as high as 8–10 times per hour, which affected his ability to fall asleep. However, there were no symptoms after the patient had fallen asleep. The symptoms were slightly relieved after hemodialysis. After a comprehensive evaluation by the nephrology department and anesthesiologist, the patient was diagnosed with RLS, with an IRLS score of 29. After the evaluation, the patient was treated 4 times with SGB, after which his symptoms were relieved. After 2 weeks, the symptoms worsened. After 2 supplementary treatments, the symptoms were relieved, and there were occasional seizures. The average sleep time was approximately 3 h earlier. One month later, the IRLS score decreased to 18. Half a year later, the patient felt that the frequency of leg twitching had increased slightly, but the symptoms could be relieved after hemofiltration. At this evaluation, the IRLS score was 11. He now felt that this condition had little impact on his normal life and did not seek further treatment. Thus far, compared with that before treatment, the patient’s sleep quality has significantly improved, and polysomnography has shown that the longest sleep pause time has been reduced.

### 2.3 Case 3

A 24-year-old female, was admitted to our hospital in August 2021 due to renal hypertension. In addition, she had a history of stage 5 chronic kidney disease, renal bone disease, and type 1 diabetes. An examination revealed that an arteriovenous fistula in the left forearm was dysfunctional, and it was treated surgically. Anticoagulation, dialysis and other symptomatic treatments were performed after the operation, and the patient’s condition was stable. Since 2020, the patient had had difficulty falling asleep at night, manifested as spontaneous twitching of the waist and legs before bed, with a frequency of approximately 5–8 times per hour, which could be relieved after stretching. Occasionally, the patient needed to walk around to get relief; and the number of awakenings at night was more than 10. After a comprehensive evaluation by the nephrology department and anesthesiologists, she was diagnosed with RLS, with an IRLS score of 31. After a full assessment of the patient, an SGB was administered 6 times, the symptoms were slightly relieved at first, and the International Respiratory Scale (IRLS) score decreased to 22 1 month later. It has been more than half a year now, and occasionally, the patient’s leg twitches; she wakes up approximately 1–2 times a month; and the IRLS score has dropped to 10. The polysomnography results showed that the patient’s sleep disorder index was significantly lower, and the proportion of time the patient spent snoring was also lower (Tables 1–3).

**Table 1:** Clinical characteristics of the patients.

Case	Case 1	Case 2	Case 3
Age (years)	47	57	24
Gender	Male	Male	Female
Smoking history	No	No	No
Drinking history	More than 20 years	No	No
History of diabetes	No	No	Type I Diabetes, 2 years
History of hypertension	17 years, Blood pressure as high as 200/110 mmHg	More than 10 years, Blood pressure as high as 180/100 mmHg	2 years, Blood pressure as high as 210/100 mmHg
Renal diseases history	More than 3 years	More than 4 years	More than 2 years
History of other diseases	No	No	No
Family history of disease	His father suffers from hypertension	No	No

**Table 2:** Patient information on stellate ganglion block treatment.

Case		Case 1	Case 2	Case 3
The start date of RLS treatment		2021.08	2021.07	2021.08
Therapy method		0.5% Ropivacaine 5 mL, alternate left and right, every other day		
Operating steps		The patient was placed in a supine position with his head was tilted to one side. Under ultrasound guidance, puncture was performed to the vicinity of the 6th cervical vertebrae and the anterior tuberosity of the transverse process to a depth of 3.0–3.5 cm. After the retrieval revealed that there was no blood, cerebrospinal fluid, or gas, 5 mL of 0.5% ropivacaine was slowly injected. After the operation, the patient was asked if there was any discomfort. The patient was asked to change to a sitting position, and he was observed as to whether there was Horner syndrome.		
Adverse reactions		No	No	No
Number of SGB treatments		6	4 + 2	6
IRLS score	Initial	34	29	31
	Followed up for 1 month	8	18	22
	Finish (2022.12)	10	11	10

**Table 3:** Patient sleep monitoring reports.

			Initial	One month	Now
Case1	Sleep percentage	Snoring	35.5%	8.1%	7.9%
		Airflow restriction	17.1%	11.0%	11.2%
		Paradoxical breathing	13.7%	0%	0%
	Disorder index		18.4/h	14.3/h	13.8/h
	Longest sleep apnea period		81.9 s	30.1 sç	35.4 s
Case2	Sleep percentage	Snoring	0.7%	5.9%	3.4%
		Airflow restriction	11.3%	15.6%	13.2%
		Paradoxical breathing	4.8%	11.0%	7.9%
	Disorder index		19.5/h	17.7/h	16.6/h
	Longest sleep apnea period		73.1 sç	53.2 s	40.8 s
Case3	Sleep percentage	Snoring	27.8%	15.7%	15.4%
		Airflow restriction	12.4%	11.9%	12.3%
		Paradoxical breathing	0%	0%	0%
	Disorder index		18.9/h	14.2/h	13.9/h
	Longest sleep apnea period		34.2 s	28.9 s	23.5 s

## 3 Discussion

RLS is a common neglected disease that requires careful observation and history-taking to make a definite diagnosis and to take further diagnostic and therapeutic measures (Kambampati et al., 2020). Similarly, the patient in our report was diagnosed with nephrotic syndrome when he was hospitalized in the nephrology department of our hospital, and the doctor invited the anesthesiologist for consultation after the patient was asked about the details of the case. Therefore, it is very important to increase the reporting of RLS, which may help us identify additional patients who meet the diagnostic criteria for this disease and take timely treatment measures.

The mechanism of action of SGB mainly involves the sympathetic nervous system, affecting both the central nervous system and the peripheral nervous system. On the one hand, it restores the body’s autonomic nervous system, endocrine system, immune system, etc. (Lipov et al., 2020). On the other hand, blocking preganglionic and postganglionic nerve fibers inhibits gland secretion and pain transmission in organs and systems dominated by sympathetic nerves in the distribution area (Noma et al., 2013). SGB has been widely used to treat pain syndrome, stress and sleep disorders (Makharita et al., 2012).

There is a correlation between the sympathetic nervous system and the development of RLS. One study showed that hypofunction of the A11 diencephalospinal pathway (a brain dopaminergic pathway that innervates preganglionic sympathetic neurons and the dorsal horn of the spinal cord) leads to increased RLS-related sensory input coupled with increased peripheral sympathetic outflow, ultimately leading to RLS symptoms (Walters and Rye, 2009). Additionally, the sympathetic nervous system plays an important role in the regulation of the immune system (Kenney and Ganta, 2014). Recent studies have shown that several RLS-related diseases are related to systemic inflammation and/or immune disorders (Tanaka and Okusa, 2020; Zhu et al., 2022). Inflammation-mediated elevation of IL-6 can lead to an increase in hepcidin, a hormone that regulates iron levels, which in turn reduces serum iron levels and induces RLS symptoms (Alaçam Köksal et al., 2023). SGB can modulate a variety of immune cells and cytokines (Lipov et al., 2020). Studies by Yang et al. have shown that SGB leads to a decrease in the concentration of proinflammatory cytokines such as IL-6 (Yang et al., 2015). Therefore, SGB may be effective at controlling symptoms of RLS and improving sleep quality.

We reported 3 patients with nephrotic syndrome requiring hemodialysis. After several SGBs, the patients’ restless leg symptoms and sleep quality improved significantly. This finding suggested that SGB is effective in patients with severe comorbidities and can provide a good long-term prognosis. Moreover, this opens up additional possibilities for RLS patients with nephrotic syndrome. For patients with nephrotic syndrome, SGBs act locally, and there is no problem with unstable blood drug concentrations during dialysis. Moreover, it can eliminate patients’ anxiety about the long-term use of drugs that will increase the burden on the kidneys and lead to aggravation of the disease.

Judging from the treatment effect, the patients’ symptoms were relieved or gradually relieved in the short term after treatment. Generally, the long-term prognosis is good, and patients are highly satisfied with the treatment effect. Although longer-term follow-up data are lacking, there may be cases of relapse over time. However, our available data still suggest that SGB holds promise as an alternative therapy in RLS patients with nephrotic syndrome.

In conclusion, SGB has broad application prospects in the treatment of patients with RLS. In the future, we will conduct randomized controlled trials to evaluate the safety and efficacy of SGB and provide experimental evidence for whether this therapy can be applied clinically.

## Data availability statement

The original contributions presented in the study are included in the article/supplementary material, further inquiries can be directed to the corresponding author.

## Ethics statement

The studies involving humans were approved by Medical Ethics Committee, Shanxi Academy of Medical Sciences, Shanxi Bethune Hospital. The studies were conducted in accordance with the local legislation and institutional requirements. The participants provided their written informed consent to participate in this study. Written informed consent was obtained from the individual(s) for the publication of any potentially identifiable images or data included in this article.

## Author contributions

YL: Writing – original draft. WenZ: Writing – review & editing. HW: Writing – review & editing. WeiZ: Writing – review & editing.
